# Sustainable dietetic models: strategies for communication and promotion: Francesca Scazzina

**DOI:** 10.1093/eurpub/ckac129.220

**Published:** 2022-10-25

**Authors:** F Scazzina, B Biasini, A Rosi, C Franchini

**Affiliations:** Department of Food and Drugs, University of Parma, Parma, Italy

## Abstract

Global Burden of Disease identifies the makeup of diets as a significant risk factor for mortality and morbidity, with 11 million deaths and 255 million disability-adjusted life years attributable to dietary risk factors. 690 million people lack sufficient food and economic projections suggest that COVID-19 pandemic may add an additional 83 to 132 million people to the ranks of the undernourished, as the outbreak has exacerbated the global food flaws and insufficiencies, impacting the most vulnerable populations. Diets and related food systems also contribute to significant environmental degradation and climate change. Demand for animal-source foods is also increasing, particularly in emerging economies, which entails risks for the environment. 1.3 billion tons of food are wasted globally yearly, utilizing 38% of total energy consumption in the global food system. The real cost of acquiring enough nutrient-rich food to meet national dietary guidelines for a healthy diet exceeds available income for ∼38% of the world's population. A balanced diet that meets food-based dietary guidelines calls for even larger quantities of more costly food groups than would be needed just for nutrient adequacy, owing to their many functional attributes beyond just the essential nutrients that they contain. Faced with this scenario, there is urgent need for an appropriate strategy to increase people's awareness of the relationship between specific food choices and health and to facilitate the educational environment on this issue. Actual examples of current strategies for communication and promotion of healthy and sustainable diets will be discussed. With awareness and knowledge, clear and precise information, a supportive social environment, available and accessible healthy and sustainable food items, and the implementation of related policies, individuals have a great potential to achieve healthiness and environmental sustainability by choosing healthier and more sustainable foods.

